# From an Introductory Lecture, Delivered by Dr. Warrington, at the Philadelphia Dispensary, March 5th, 1839

**Published:** 1839-07-06

**Authors:** 


MEDICAL EXAMINER.

DEVOTED TO MEDICINE, SURGERY, AND THE COLLATERAL SCIENCES.

No. 27.] PHILADELPHIA, SATURDAY, JULY 6, 1839. [Vol. II.

*From an Introductory Lecture, delivered by* Dr. Warrington, *at the Philadelphia Dispensary, March 5th*, 1839.

This Institution, within the hall of which we are now assembled, is dedicated to the medical relief of respectable persons, though in humble circumstances, in those cases where removal to a public hospital would for any approved reason be ineligible.

It was established at the instance of Dr. Samuel Powell Griffitts, at one time Professor of Materia Medica in the University of Pennsylvania, associated with several other medical men, as well as a very respectable body of citizens. Amongst them was the late venerable Bishop White, who, from its foundation till the period of his death, more than fifty years, presided over it with a zealous care, and under whose administration, aided by an efficient board of managers, with the lamented Dr. Griffitts as secretary, for more than forty years, its fiscal and medical concerns have prospered, until more than 145,000 cases have been prescribed for, and attended upon by the physicians of the Institution.

The operations of this Dispensary extend to the entire limits of the city, which is divided into six districts, over each of which a medical officer is appointed to tend upon and prescribe for such cases of disease as may apply at this central office.

In each district the prescriber has thus an opportunity of witnessing every variety of disordered action, whether of an acute or chronic, of a mild or malignant character, occuring from the earliest infancy to the extremest old age.

A number of patients who are able to walk, come to the office of the Dispensary at stated periods to be prescribed for. This part of the service is done by two of the physicians at the same time, one of whom receives the patients from the city, north of the whole line of Chesnut street, from three to four o'clock, on alternate days of the week, except the Sabbath, while the other attends on the intervening days, to receive those who come from the south part of the city.

By this kind of organization, from 3,000 to 5,000 patients obtain professional advice and attendance every year, making an average of more than 500 cases under the charge of each physician.

At the office of the establishment, the prescriptions are compounded by the apothecary or his assistant. Orders are executed for bleeding and cupping such patients as are able to come to the house, while, in the event of disability, the directions for these remedies are attended to in the chamber of the patient.

Numerous dental operations, especially the extraction of teeth, are also performed at the office of the Dispensary.

As a very large number of the subjects who partake of the benefits of this charity, are necessarily confined in small rooms, narrow and ill ventilated alleys, in which the population is thickly crowded, they are exposed to the most potent influences of epidemic or contagious diseases, and a variety of phenomena which escape the eye of the private practitioner, or hospital physician, are almost constantly presented to the officers of this Institution, while opportunities for autopsies in the diseases of various ages, are sufficiently numerous to aid in determining morbid changes, and thus giving greater precision in the diagnosis.

In the original plan of the Dispensary organization, all cases, whether acute or chronic, surgical or obstetrical, were to be attended to by the six medical officers, aided, as occasion might require, by one or more consulting physicians or surgeons; but, by a somewhat recent arrangement, a special department of obstetrics has been established, and the pregnant and parturient females placed under the charge of your lecturer, with the intention to found a clinical school of practical midwifery,—in which the advanced student should be allowed to attend upon parturient and puerperal cases, to witness the changes which occur during actual labour, and by such opportunities, added to repeated demonstrations and manipulations upon obstetric models, instruments, &c., as well as frequent preparations of the bed for delivery, the student, or the recent graduate, may become prepared to enter into responsible practice, in the location he has selected for the exercise of his profession.

While the department of midwifery then will peculiarly occupy the attention of your lecturer, he feels that he would not discharge his duty to those who are ardently engaged in preparing themselves to become ministers of health to their fellow beings, without calling the attention of the medical classes who assemble in this city, to the invaluable opportunities which this extensive and ancient charity affords, as a school of clinical instruction in the various departments of practical medicine.

Already has the experiment been tried to the satisfaction of the pupils and teacher in the general practice; and already has the enterprise of your lecturer been so far appreciated, that a considerable number of gentlemen have attended upon the instructions and practice of midwifery, with an ardour which, I trust, gives an earnest of their future usefulness.

Since my connexion with this institution, about the middle of 1837, 90 deliveries have occurred, at nearly all of which some one or more of my pupils were present.

So important is the subject of obstetrics considered by the French government, that at the Maternity at Paris two lectures are given by a professor every week, on the theory of obstetrical science, one practical lesson daily by the midwife-in-chief, a recapitulation every evening by the most advanced pupil, and an occasional examination by some one of the corps on the amount of knowledge thus acquired. The pupils are then allowed to attend upon cases of labour, at which the principal or an expert presides,—and in this way the pupils become theoretically and practically qualified for the exercise of a responsible profession. If, in old countries, whose institutions have become consolidated by the experience of ages, such arrangements are found necessary, can our own present a better plan? Will oral instructions, accompanied by a single demonstration of the rules of practice upon a model, ever be carried out so perfectly as to prove sufficient substitutes for practical information, which can alone be obtained by a diligent attendance upon obstetric cases under the guidance and direction of one familiar with the practice of midwifery? To supply, in some degree, the necessity of the case, your lecturer, after much reflection, determined upon the arduous enterprise of opening the avenues of this extensive charity for the improvement of such gentlemen as were disposed to avail themselves of opportunities to attend upon a clinical course of obstetrics.

The plan of instruction, as has been already announced, consists of familiar, or colloquial lectures, of demonstrations of the pelvis, and such particulars as relate to parturition, a demonstration of the anatomy of the organs of conception, gestation, and parturition, history of the development of the fœtus, the progress of gestation, history of labour, preparation of the bed for delivery, the arrangement of the patient for this purpose, description of the different changes which the os uteri undergoes during labour, general duty and conduct of the accoucheur during this time, the detection of the presentation and positions of the fœtus at the different stages of labour, the attentions necessary for the child and mother at the time of and subsequent to birth, the various manipulations upon the mannekin, for the purpose of becoming familiar with the operation of turning, and with the rules and experiments for the application of forceps. These instructions, therefore, will be strictly practical; and it is contemplated that each member of the class will have the opportunity of repeating them till he will feel conscious of a familiarity which will relieve him from much embarrassment he would otherwise experience, when called upon to use his knowledge on any emergency.

As the lecturer has usually under his charge a number of obstetric cases in connection with this dispensary, the gentlemen who become associated with him in this course of instructions, and who have already attended a full course of lectures on midwifery, or a course of practical instructions by the accoucheur, shall be allowed to attend upon these cases, if desired, in the order in which their names are registered in the class-book.

The number of cases which apply for the benefits of this institution has been steadily increasing since my election in the middle of 1837,—and we are disposed to believe that if the duties of this department continue to be faithfully administered, in the manner originally intended by the incumbent, the confidence of the patients and community will be continued inviolate.

